# The comparison of MRN, electrophysiology and progression among typical CIDP and atypical CIDP subtypes

**DOI:** 10.1038/s41598-020-73104-1

**Published:** 2020-10-07

**Authors:** Yuan Feng, Yu Zhang, Xiaoyun Su, Chuansheng Zheng, Zuneng Lu

**Affiliations:** 1 Department of Neurology, Renmin Hospital of Wuhan University, Wuhan, 430060 Hubei Province People’s Republic of China; 2 Department of Hyperbaric Medicine, 6th Medical Center of PLA General Hospital, Beijing, People’s Republic of China; 3 Department of Radiology, Union Hospital of Tongji Medical College, Huazhong University of Science and Technology, Wuhan, 430022 Hubei Province People’s Republic of China

**Keywords:** Immunology, Diseases, Medical research, Neurology

## Abstract

We aimed to compare the electrophysiology and magnetic resonance neurography (MRN) results of chronic inflammatory demyelinating polyradiculoneuropathy (CIDP) subtypes and to explore the progression from atypical CIDP to typical CIDP. We collected the medical records of 45 CIDP patients to analyse the rate of progression from atypical CIDP to typical CIDP subtypes. The cerebrospinal fluid (CSF) protein (p = 0.024) and overall disability sum score (ODSS) (p = 0.000) differed among patients with typical CIDP, distal acquired demyelinating symmetric neuropathy (DADS) and Lewis-Sumner syndrome (LSS). The compound motor action potential (CMAP) of typical CIDP was lower than that of the other subtypes (p = 0.016, p = 0.022 and p = 0.012). The cross-sectional area (CSA) of nerve roots in typical CIDP was significantly thicker than that of nerve roots in DADS and LSS. There were fewer DADS and LSS patients who progressed to typical CIDP than those who progressed to pure motor and pure sensory CIDP (p = 0.000), and the progression from pure motor to typical CIDP required a significantly longer time than the progression from pure sensory to typical CIDP (p = 0.007). Typical CIDP was more severe than the other subtypes not only in terms of clinical and electrophysiology factors but also in terms of MRN factors.

## Introduction

Chronic inflammatory demyelinating polyradiculoneuropathy (CIDP) is a rare immune-mediated disease that targets the myelin sheaths of peripheral nerves; this disease has a chronic course and often causes disabled sensory-motor neuropathy^1^. CIDP was divided into typical and atypical CIDP by the Joint Task Force of the European Federation of Neurological Societies and the Peripheral Nerve Society (EFNS/PNS)^2^. The clinical presentation of typical CIDP includes chronically progressive or recurrent symmetric proximal and distal weakness, sensory dysfunction, and absent or reduced tendon reflexes in all extremities^3^. Atypical CIDP is regarded as a clinical variant of CIDP and is classified into five subtypes according to various clinical symptoms: distal acquired demyelinating symmetric neuropathy (DADS), pure motor or sensory CIDP, Lewis-Sumner syndrome (LSS) and focal CIDP^4,5^. The different CIDP subtypes have different responses to treatment^6,7^.

There is an interesting phenomenon regarding typical and atypical CIDP, where the diagnosis is not fixed but can change over time. For example, some patients may initially present with pure sensory, pure motor, or LSS that then evolves over a few months to a typical sensorimotor form^8,9^. Another point that should be noted is that there is no clear boundary with delineated criteria for the diagnosis of atypical CIDP. For example, it is unclear whether CIDP should include patients with clinical manifestations of sensory impairments but both sensory and motor neuro-electromyography abnormalities or those only with clinical and electrophysiology sensory disturbances^4^.

The different subtypes of CIDP have not only different clinical features and treatment responses but also diverse imaging and electrophysiology performances^10–12^. The aim of our study is to prospectively explore the discrimination of typical CIDP from its variants according to electrophysiology and magnetic resonance neurography (MRN). At the same time, we retrospectively researched the features and frequencies of atypical CIDP conversion to typical CIDP.

## Results

### Clinical features

A total of 32 typical CIDP patients (mean age 50.00 ± 14.38 years, 8 women), 6 DADS patients (mean age 45.8 ± 10.47 years, 2 women) and 5 LSS patients (mean age 39.0 ± 12.12 years, 1 woman) underwent clinical, electrophysiological, and MRN evaluations. We only compared the data of typical CIDP, DASD and LSS patients because the number of pure motor and sensory CIDP patients was too low for the analysis. The demographic and baseline clinical data are presented in Table 1. The data regarding sex, age, onset age and disease duration were not significantly different among the three groups, but the values of cerebrospinal fluid (CSF) protein and the overall disability sum score (ODSS) in typical CIDP patients were higher than those in DADS and LSS patients.

**Table 1:** Comparison of clinical features in typical CIDP, DADS and LSS.

Parameters	Typical CIDP (n = 32)	DADS (n = 6)	LSS (n = 5)	P value
Sex (M/F)	24/8	4/2	4/1	0.871
Age (years)	47.00 (38.00,59.00)	39.50 (36.75,56.00)	45.00 (24.50,46.00)	0.098
Age at disease onset (years)	42.00 (34.75,55.25)	37.50 (34.00,51.25)	41.00 (23.50,42.00)	0.191
Disease duration (months)	39.00 (21.75,63.00)	30.00 (20.25,45.00)	36.00 (18.50,46.50)	0.518
CSF protein (g/L)	0.90 (0.55,1.11)	0.56 (0.48,0.59)	0.35 (0.32,1.06)	*0.024*
ODSS grades	3 (2,4)	2 (1,2)	1 (1,2)	*0.000*

Italic indicates the p < 0.05.

*CIDP* chronic inflammatory demyelinating polyradiculoneuropathy, *DADS* distal acquired demyelinating symmetric neuropathy, *LSS* Lewis-Sumner syndrome, *CSF* cerebrospinal fluid.

### Electrophysiological comparison

The CMAPs of the median nerves in DADS patients and the CMAPs of the tibial and peroneal nerves in LSS patients were all higher than those in typical CIDP patients (p = 0.016, p = 0.022 and p = 0.012). The SCVs of the median and ulnar nerves in LSS patients were slower than those in typical CIDP (p = 0.004 and p = 0.001) and DADS (p = 0.000 and 0.007) patients. Although the F-wave latency of tibial nerves was long in typical CIDP patients, a definite F-wave was absent in some patients, which was especially serious in typical CIDP patients, whose 19 (29.69%) median nerves, 28 (43.75%) ulnar nerves and 33 (51.56%) tibial nerves were absent. For DADS patients, 3 (25%) median nerves, 2 (16.67%) ulnar nerves and 4 (33.33%) tibial nerves were absent, while for LSS patients, the F-waves of all nerves could be drawn. There was a significant discrepancy in the frequency of F-wave absence among typical CIDP, DADS and LSS patients in the ulnar and tibial nerves (p = 0.006 and 0.003) (Table 2).

**Table 2:** Comparison of bilateral nerve electromyography in typical CIDP, DADS and LSS.

	Typical CIDP (n = 64)		DADS (n = 12)		LSS (n = 10)		P value
	Median	Q1–Q3	Median	Q1–Q3	Median	Q1–Q3	
**Median nerve**							
MCV (m/s)	42.80	28.90–53.15	45.50	36.90–46.00	46.05	45.43–55.15	0.175
CMAP (mV)	4.30	2.28–7.20	7.20	3.60–9.50	7.45	6.00–9.03	0.016*
F-Lat (ms)	35.70	30.60–49.55	32.20	28.75–35.45	33.90	32.68–34.63	0.256
SCV (m/s)	51.00	43.00–61.20	55.50	50.50–61.95	40.25	34.50–44.13	*0.004*^*#*^, *0.001*^*&*^
SNAP (uV)	14.20	10.83–23.10	18.00	14.65–36.25	13.45	11.38–15.60	0.075
**Ulnar nerve**							
MCV (m/s)	43.50	33.33–58.80	44.55	34.10–47.43	43.90	42.98–46.58	0.852
CMAP (mV)	5.40	3.16–7.65	5.00	4.43–11.75	9.05	6.63–10.78	0.072
F-Lat (ms)	33.70	28.85–42.08	33.60	32.08–41.78	34.75	33.60–37.70	0.638
SCV (m/s)	56.00	44.25–61.25	55.30	46.50–58.20	41.85	33.43–42.70	*0.000*^*#*^*,0.007*^*&*^
SNAP (uV)	15.00	9.15–22.05	13.00	12.70–35.05	10.15	8.10–12.60	0.105
**Radial nerve**							
SCV (m/s)	56.60	44.38–68.68	55.30	50.90–69.00	42.65	38.63–52.53	*0.040*^*#*^
SNAP (uV)	14.20	10.83–23.10	14.10	13.20–31.70	16.05	15.30–22.33	0.447
**Tibial nerve**							
CMAP (mV)	2.45	0.69–11.28	9.25	0.50–12.15	10.70	6.75–14.15	*0.022*^*#*^
F-Lat (ms)	56.00	50.00–70.40	63.95	62.48–68.85	57.90	55.50–59.00	0.038*
**Peroneal nerve**							
MCV (m/s)	40.00	31.85–42.98	34.40	24.55–36.03	36.20	35.00–36.85	0.140
CMAP (mV)	2.35	0.26–4.18	4.75	0.23–5.13	4.35	3.28–13.50	*0.012*^*#*^
**Sural nerve**							
SCV (m/s)	45.00	38.00–51.90	44.00	41.00–61.30	37.15	36.30–43.58	0.194
SNAP (uV)	10.50	7.20–14.80	3.10	3.00–21.90	13.35	10.75–13.93	0.326

Italic indicates the p < 0.05.

*MCV* motor nerve conduction velocity, *CMAP* compound motor action potential, *F-Lat* F-wave latency, *SCV* sensory nerve conduction velocity, *SNAP* sensory nerve action potential.

*Typical CIDP compared with DADS.

^#^Typical CIDP compared with LSS.

^&^DADS compared with LSS. Q1 = the first quartile; Q3 = the third quartile.

### Cross-sectional area comparison

**Figure 1:**
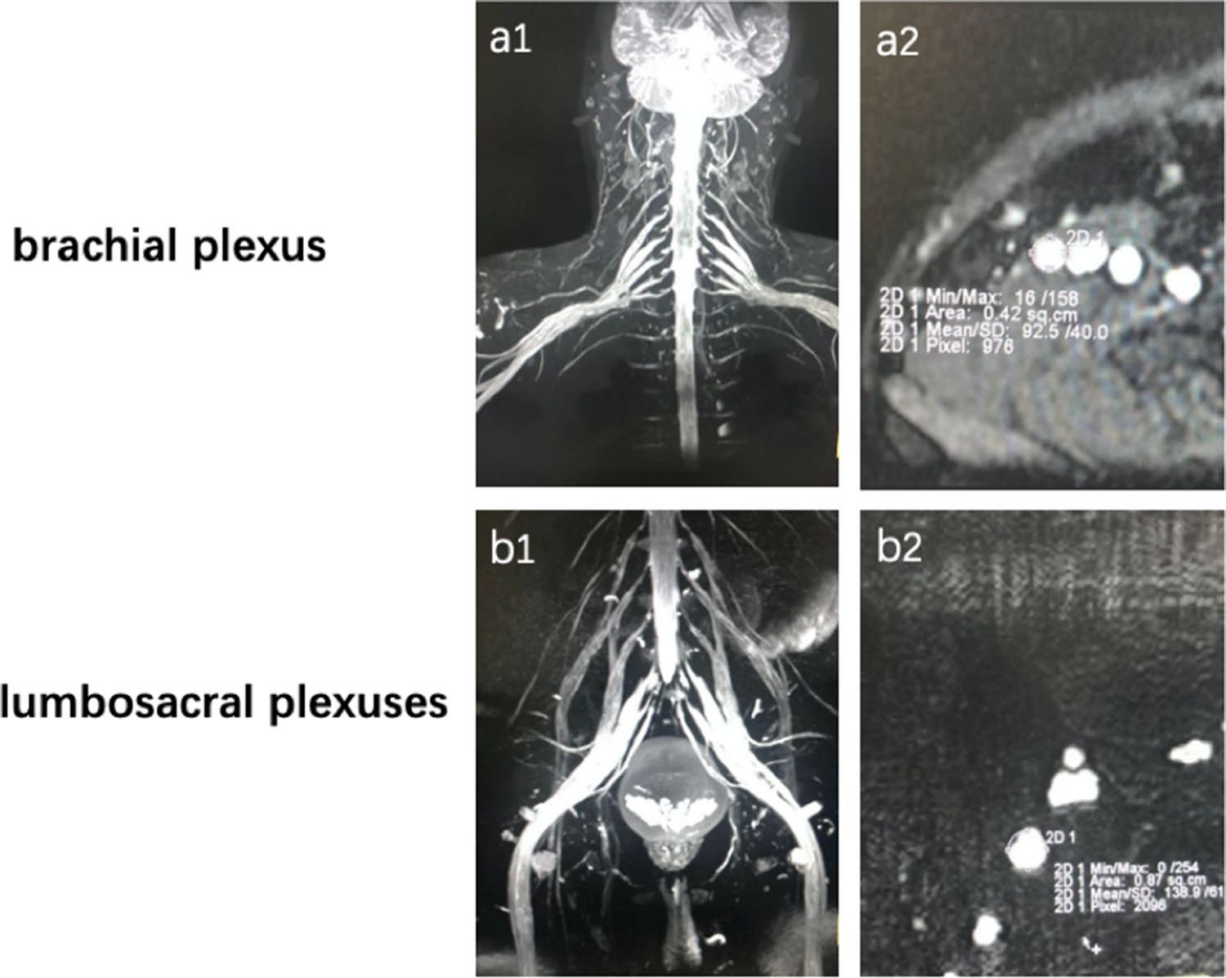
Representative MRN of typical CIDP patients. (**a**) MIP reconstruction of the brachial plexus and the C8 cross-sectional area. (**b**) MIP reconstruction of the lumbosacral plexus and the S1 cross-sectional area.

The CSA data of the brachial and lumbosacral plexuses in typical CIDP, DADS and LSS patients are presented in Table 3. We measured both sides of the CSA of the brachial and lumbosacral plexus roots in each patient. The CSAs of C7, C8, L4, L5, and S1 in typical CIDP patients were significantly thicker than those in DADS and LSS patients, and there was no obvious difference between DADS and LSS patients in terms of the CSAs (Fig. 1).

**Table 3:** Comparison of the nr-CSA of the brachial and lumbosacral plexuses in typical CIDP, DADS and LSS.

nr-CSA	Typical CIDP (n = 64)		DADS (n = 12)		LSS (n = 10)		P value
	Median	Q1–Q3	Median	Q1–Q3	Median	Q1–Q3	
C7	32.68	25.86–47.15	21.76	18.39–33.73	21.10	19.93–27.98	0.006*, *0.003*^*#*^
C8	33.55	23.42–48.43	24.60	20.28–28.72	22.25	21.60–23.10	0.037*, *0.015*^*#*^
L4	47.09	29.85–76.20	26.84	24.16–30.98	24.30	22.65–27.76	0.006*, *0.001*^*#*^
L5	70.85	46.88–113.90	47.11	37.69–58.32	37.60	27.75–42.75	0.045*, *0.000*^*#*^
S1	74.00	40.95–102.38	43.39	38.97–50.91	38.00	29.48–45.00	0.048*, *0.004*^*#*^

Italic indicates the p < 0.05.

nr-CSA, nerve root cross-sectional area.

*Typical CIDP compared with DADS.

^#^Typical CIDP compared with LSS.

^&^DADS compared with LSS.

### The progression of CIDP subtypes

**Figure 2:**
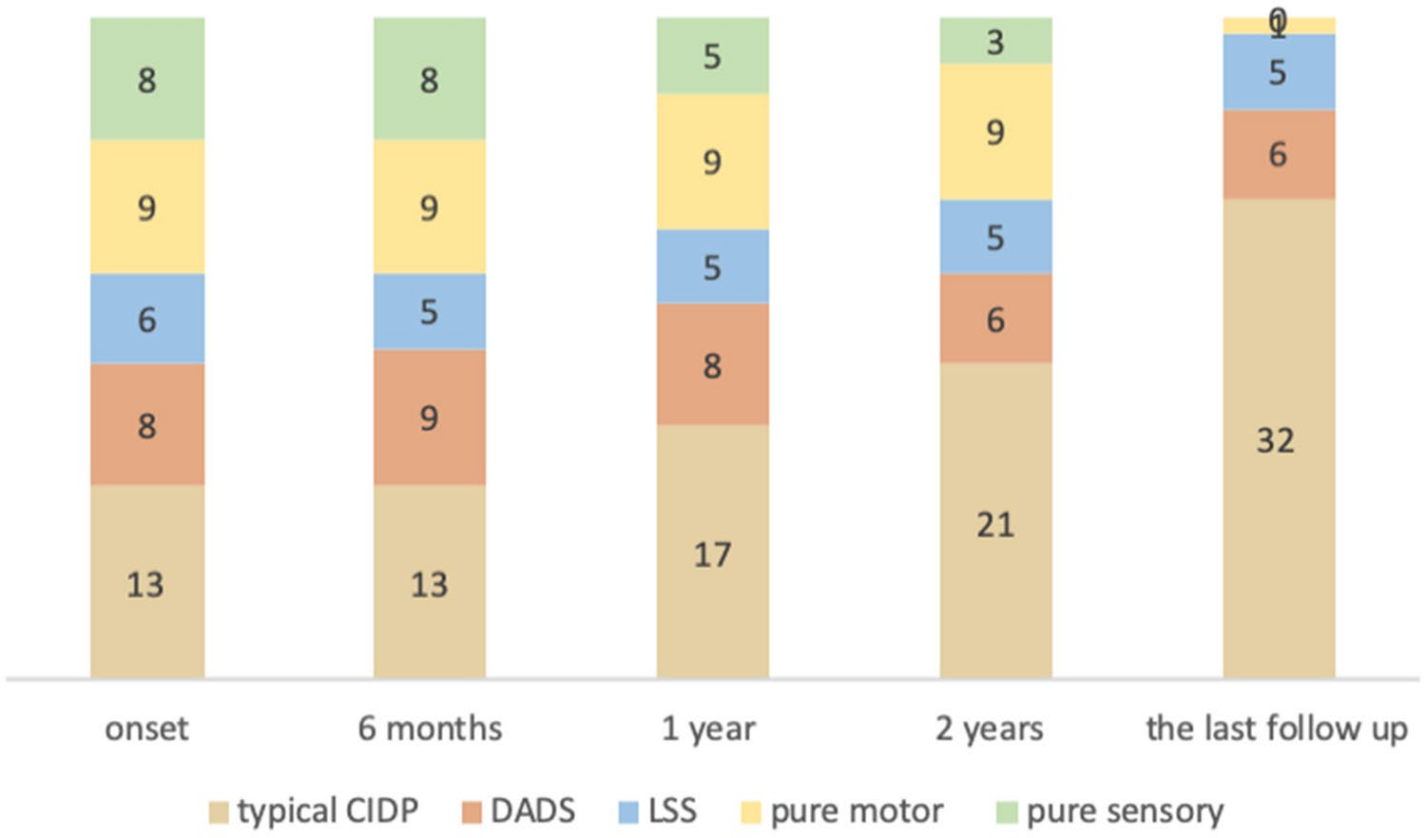
The progression of CIDP subtypes at appointed time points. The progression of CIDP subtypes at onset, 6 months, 1 year, 2 years and the last follow-up is displayed as the number of patients.

As a retrospective-prospective study, we reviewed the patients’ symptoms at the time of onset and classified them as typical CIDP or subtypes of atypical CIDP; then, we recorded the changes in types according to the progression of the disease. Only 13 patients met the EFNS/PNS criteria for typical CIDP at the beginning, but the number of patients increased to 18 after one year and to 32 at the end of study after a mean disease duration of 4 (2, 6) years; the additional 19 typical CIDP patients were converted from 2 DADS, 1 LSS, 8 pure motor and 8 pure sensory patients. Eight patients had a diagnosis of DADS at the time of onset, whose symptoms and signs presented symmetrically in the upper (1 case) or lower (7 cases) distal limbs; then, 2 (25%) of them progressed to typical CIDP 1 and 5 years later, and the other patients still had DADS after a duration of 2.5 (1.7, 3.8) years. Six patients were diagnosed with LSS at disease onset, but the diagnosis was changed to DADS in only 1 (16.67%) patient half a year later; the diagnosis was then changed to typical CIDP 1 year later, and the other 5 patients still had DADS after 3 (1.5, 3.9) years. Furthermore, 8 of 9 (88.89%) pure motor CIDP patients progressed to typical CIDP after 5.25 (2.02, 11.38) years, and all 8 (100%) pure sensory CIDP patients progressed to typical CIDP 0.96 (0.65, 2.02) years later. Figure 2 indicates that there were fewer DADS and LSS patients who progressed to typical CIDP than those who progressed to pure motor and pure sensory CIDP patients (p = 0.000), but the difference between pure motor and pure sensory CIDP was that the former progressed to typical CIDP and required a significantly longer time than the latter (p = 0.007).

## Discussion

CIDP is classified as typical and atypical CIDP variants according to different definitions^13–19^. In this study, we applied the diagnostic criteria for EFNS/PNS and explored the comparison of clinical features, MRN, electrophysiology and progression for different CIDP subtypes, as very little research exists in the literature^20,21^. Our study showed that the values of CSF and ODSS in typical CIDP patients were higher than those in DADS and LSS patients. MRN and electrophysiology play important roles in the diagnosis of CIDP and in the differential diagnosis of CIDP and other peripheral neuropathies. Although CSA and CSF were different in CIDP patients with typical and atypical variants, disease severity (higher in typical CIDP) may be an important confounder that would need to be addressed in larger studies with multivariate analysis^22–24^. The results of this study indicated that the SCVs of the median and ulnar nerves in LSS patients were slower than those of typical CIDP and DADS patients, and the nerve root CSAs of the brachial and lumbosacral plexuses in typical CIDP patients were significantly thicker than those in DADS and LSS patients. To assess the progression of the disease, we retrospectively classified patients according to the symptoms at the time of disease onset and then recorded the rate of progression from atypical CIDP subtypes to typical CIDP at certain times. We found that fewer DADS and LSS patients progressed to typical CIDP patients than pure motor and sensory CIDP patients and that pure sensory CIDP patients progressed to typical CIDP faster than pure motor CIDP patients.

It has been reported that patients with DADS have an older onset age than CIDP patients, and other subtypes were not significantly different when compared with typical CIDP in a large database study of Italian CIDP patients; these results were in contrast to the results of a Japanese study that included 139 CIDP patients in whom LSS patients were younger than typical CIDP patients^7,20^. The outcome of our study was that age, onset age, duration and were not different among typical CIDP, DADS and LSS patients, which was the same as the results of a recent report from Nagoya University, Japan^25^. These differences in several studies might be caused by the use of different phenotypic standards or the different geographical locations, but a similar result was that we all found that typical CIDP patients had more serious disability scores than DADS and LSS patients. Currently, only two Japanese studies have compared the electrophysiology results of typical and atypical CIDP, but these studies only analysed the median, ulnar and tibial nerves, whereas our data included the addition of the radial, peroneal and sural nerves and not only included motor conduction but also sensory conduction^20,25^. Our study revealed that the MCVs of the nerves showed no significant differences among the groups, and the CMAPs of almost all typical CIDP nerves were the lowest compared with those of the other groups, which was consistent with the previous series. Our findings also showed that the rate of absent F-waves in typical CIDP patients was highest; thus, the finding that the F-wave latency of the tibial nerve in DADS patients’ was longer than that in typical CIDP patients was not accurate, as shown in the data. In accordance with a previous report, the SCVs of typical CIDP nerves were slower than those of other nerves, which was contradictory to our finding that the SCVs of LSS nerves were the slowest^25^. In terms of the reason for this discrepancy, we consider the difference in patients’ essential characteristics to be important. As these studies all have small sample sizes and as the age, sex, and disease duration of patients all have significant differences, the results will have bias according to the different clinical features. To the best of our knowledge, no papers have compared the CSAs of nerve roots among CIDP subtypes. In our study, the CSAs of nerve roots in the typical CIDP group were significantly larger than those in the DADS and LSS groups, which indicated that inflammation and demyelination of proximal peripheral nerves were more serious in the typical CIDP group than in the other groups. To assess the progression of atypical CIDP, we retrospectively analysed the symptoms at the onset of disease and during the following years, and we found that 70.45% (31/44) of patients first had atypical CIDP and that 61.29% (19/31) of them had progressed to typical CIDP at the end of the follow-up, 3.33 (1.83, 5.0) years later, a result that was similar to a previous study^8^. The rates of DADS and LSS (25% and 16.67%) progression to typical CIDP were significantly lower than those of pure motor and pure sensory progression (88.89% and 100%) after a mean disease duration of 3.3 years. A similar finding exists between this study and a previous Italian study: we both found that the highest progression rate was for pure sensory CIDP and that DADS showed a lower rate of progression, while the difference between the studies was that the Italian study reported rates of 24% for DADS, 36% for LSS, 32% for pure motor and 48% for pure sensory in terms of the progression to typical CIDP in 5 years, and the percentage values at 10 years were 39%, 63%, 64%, and 77% ^8^. We believe that this difference may be caused by the following three reasons. First, we had different follow-up times and numbers of volunteers, and it is clear that the progression rate was associated with the disease duration. Second, the ethnical differences and genetic backgrounds between Italy and China may represent reasons for the different rates of disease progression. Finally, several studies have shown that different treatment initiation times and treatment regimens may also lead to different results^26–28^. The uneven distribution of medical resources and limited therapeutic levels in China lead to the late start of treatment for patients, and most patients are treated with hormones; only a few patients have received a single course of IVIg (intravenous immunoglobulin). It is possible that the unequal medical level caused our atypical CIDP patients to progress to typical CIDP faster and more frequently.

Our results showed that the disability scores and the CMAPs of nerves were lowest in typical CIDP patients and that the CSAs of nerve roots in this group were larger than those in the other groups, which indicated that typical CIDP was more severe than other subtypes not only in terms of clinical and electrophysiology factors but also in terms of MRN factors; this also suggested that peripheral nerve damage in the typical CIDP group was most serious not only in proximal nerve roots but also in distal nerves. One phenomenon that should be pointed out is that we defined patients as having pure motor or pure sensory CIDP according to their symptoms, while the electrophysiology results of 8 pure motor and 8 pure sensory patients who progressed to typical CIDP showed both sensory and motor involvement; however, the electrophysiology results of the 1 pure motor patient who did not progress to typical CIDP showed that only motor nerves were affected. It is worth considering whether the classified criteria for subtypes should be strict, such as defining them not only by the symptoms but also by the performance of electrophysiology.

There are several limitations in our study. First and foremost, the main limitations were that it was a study with a small sample size in the validation set, and bias was difficult to avoid. The incidence rate for CIDP is 0.33 per 100,000 person-years, and the 5 subtypes of atypical CIDP account for only 18% of all CIDP patients, which increases the difficulty of data collection^29,30^. The low incidence of variant CIDP is an irreversible fact, and we are currently unable to collect more medical records. Second, we did not discuss the treatment responses for different subtypes. As a retrospective-prospective single-centre small-sample study, we retrospectively collected medical records and prospectively measured patients’ electrophysiology and MRN data. In terms of the treatment, our study did not provide any intervention, so it is not appropriate to evaluate the effects of various treatments. As far as we know, many large prospective studies have evaluated and discussed the advantages of these treatment effects^29–31^.

In conclusion, despite the limitations of the study, our data indicated that typical CIDP was more severe than other subtypes not only in terms of clinical and electrophysiology factors but also in terms of MRN factors. Pure motor and pure sensory CIDP were more inclined to progress to typical CIDP than DADS and LSS, and the progression rate of pure sensory CIDP was faster than that of other subtypes. Finally, we suggest that classified criteria should include not only symptoms but also electrophysiology results.

## Materials and methods

### Patients

Our study was a retrospective-prospective study of a consecutive series of 45 CIDP patients collected in the clinic and during hospitalization at Renmin Hospital of Wuhan University from January 2016 to May 2019. We used the electrodiagnostic criteria of EFNS/PNS for the diagnosis of CIDP, and we subclassified the patients into typical and atypical CIDP patients based on the clinical criteria in order to perform a retrospective analysis of the diagnosis at onset and of the progression throughout the course of disease^2^. Of the 45 patients, 32 had typical CIDP, 6 had DADS, 5 had LSS, and 1 had pure motor CIDP at the beginning of our study. The number of pure motor and sensory CIDP patients was too low to analysis in the prospective part, so they were just be mentioned in the retrospective part. The ODSS was used as a disability score for the clinical assessment ranging from 0 (“no signs of disability”) to 12 (“most severe disability score”).

Written informed consent was obtained from all patients before participation. The Clinical Research Ethics Committee approved this prospective study at Renmin Hospital of Wuhan University (2017K-045), and all procedures were performed following the relevant guidelines/regulations in the Declaration of Helsinki.

### Electromyography

Conduction studies and F-wave evaluations of the median, ulnar, tibial, radial, peroneal, and sural nerves were performed with standard surface stimulation and recording techniques by a Keypoint4 electromyograph from Medtronic (Denmark). The patients were lying flat on the examination bed, and the skin temperatures were maintained above 32 °C in the limbs. The parameters measured included the motor nerve conduction velocity (MCV), compound motor action potential (CMAP), F-wave latency (F-Lat), sensory nerve conduction velocity (SCV), and sensory nerve action potential (SNAP). Electrophysiology was performed in patients by two professional doctors, and all procedures fulfilled the criteria of EFNS/PNS for CIDP.

### Imaging technique

All participants were prospectively examined with a 3.0 T MR scanner (Magnetom Trio, Siemens Healthcare, Erlangen, Germany) using three-dimensional sampling perfection with application-optimized contrast and different flip angle evolution (3D SPACE) sequences with a neck matrix coil, and three-body matrix anterior coils were applied. Subjects were placed in the gantry in the supine position with the head in the neutral position and instructed to breathe normally. The contrast agent (0.1 ml/kg, Gadovist; Bayer Pharma AG) was intravenously administered before the brachial and lumbosacral plexuses were enhanced by scanning. The 3D SPACE parameters were as follows: TR/TE = 3000/270 ms, FOV = 448 × 448 mm2, voxel size = 1.0 × 1.0 × 1.0 mm^3^, slice thickness = 1.0 mm, slice gap = 0 mm, and slice = 144. The acquisition time for imaging of the brachial and lumbosacral plexuses was 20 min.

Maximum intensity projection (MIP) images were reconstructed by built-in 3D postprocessing software (3D Syngo MR workspace; Siemens Healthcare, Erlangen, Germany). The bilateral cross-sectional area (CSA) of the nerves at the C7-C8 and L4-S1 levels was measured on the coronal plane. All work was completed independently by two senior radiologists blinded to all of the patients’ information, and the CSA (nr-CSA) of the nerve roots on each side of the brachial and lumbosacral plexuses was calculated separately.

We compared the electrophysiology and MRN results of the nerve roots in the plexuses in typical and atypical CIDP patients and assessed the frequency of progression from atypical CIDP to typical CIDP.

### Statistical analysis

Statistical analysis was performed in SPSS 21.0 (SPSS, Chicago, Illinois). Values conforming to a normal distribution are shown as the mean ± standard deviation (SD); otherwise, they are expressed as the medians and quartiles (Q1, Q3). Nonparametric tests (Kruskal–Wallis test) and Bonferroni correction were used for continuous variables, and chi-square tests were used for categorical variables. Two-sided P values were calculated for all analyses. P < 0.05 was considered significant.

## Data Availability

The datasets generated during and analysed during the current study are available from the corresponding author upon reasonable request.
